# Supplementary figures and images for: Hormonally responsive bovine oviductal organoids recapitulate native oviductal secretions and enhance sperm capacitation
Source: Cell Mol Life Sci. 2026 Apr 21;83(1):223. doi: 10.1007/s00018-026-06200-6 (PMC13190940; doi:10.1007/s00018-026-06200-6)

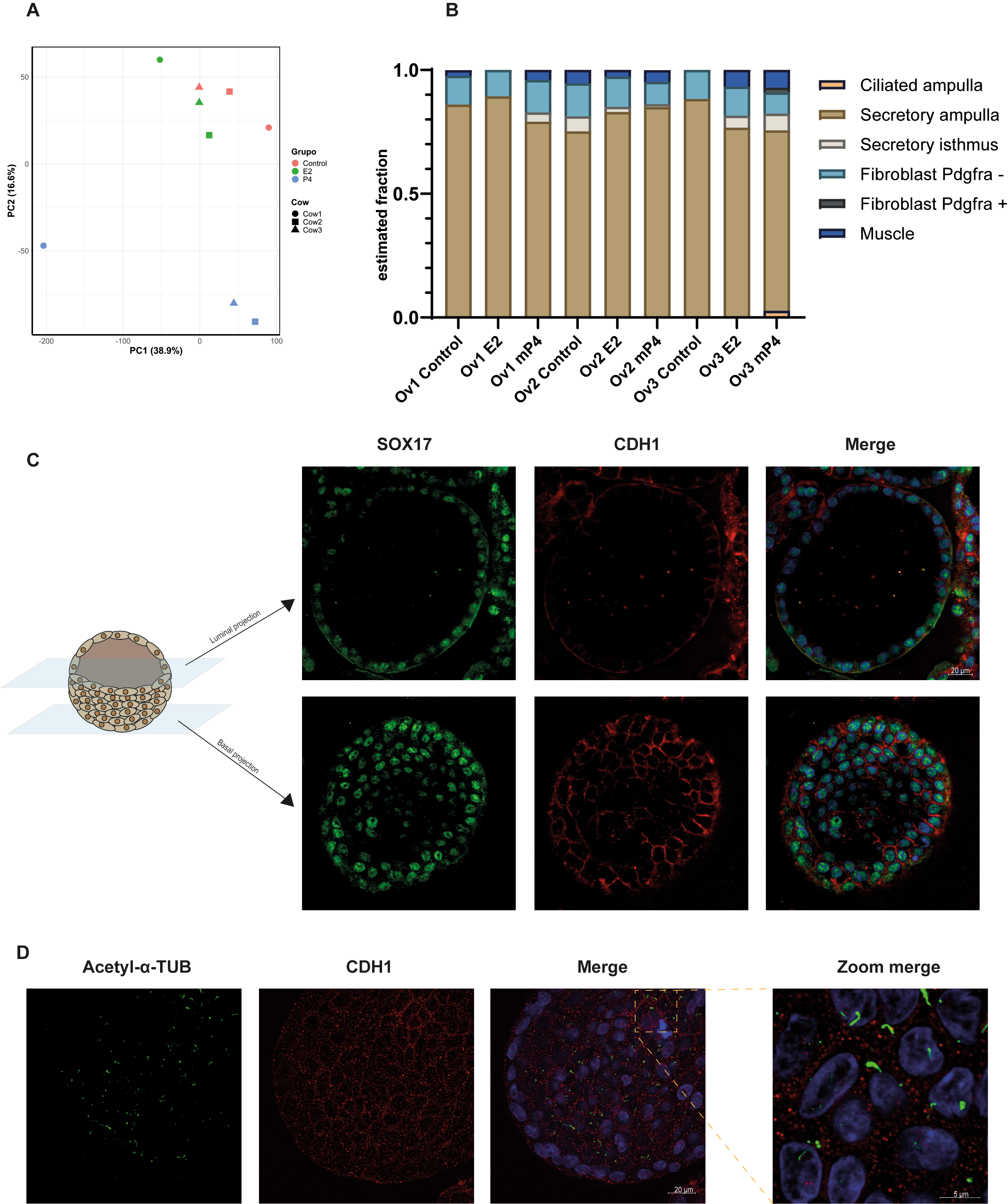

Supplement: Supplementary file 9 — Supplementary Figure 1. (A) Principal component analysis (PCA) of bulk RNA-seq data from control organoids and organoids treated with E2 or mP4. (B) Bar graph showing the estimated fractions of cell types in bovine oviductal organoids after deconvolution using singlecell RNA-seq data. (C) Immunofluorescence staining of oviductal organoids showing basal and luminal projections, stained for SOX17 (green, secretory marker), CDH1 (red), and DAPI (blue). (D) Immunofluorescence staining of oviductal organoids stained for acetyl-α-TUBULIN (green), CDH1 (red), and DAPI (blue) [file 18_2026_6200_Fig5_ESM.png]

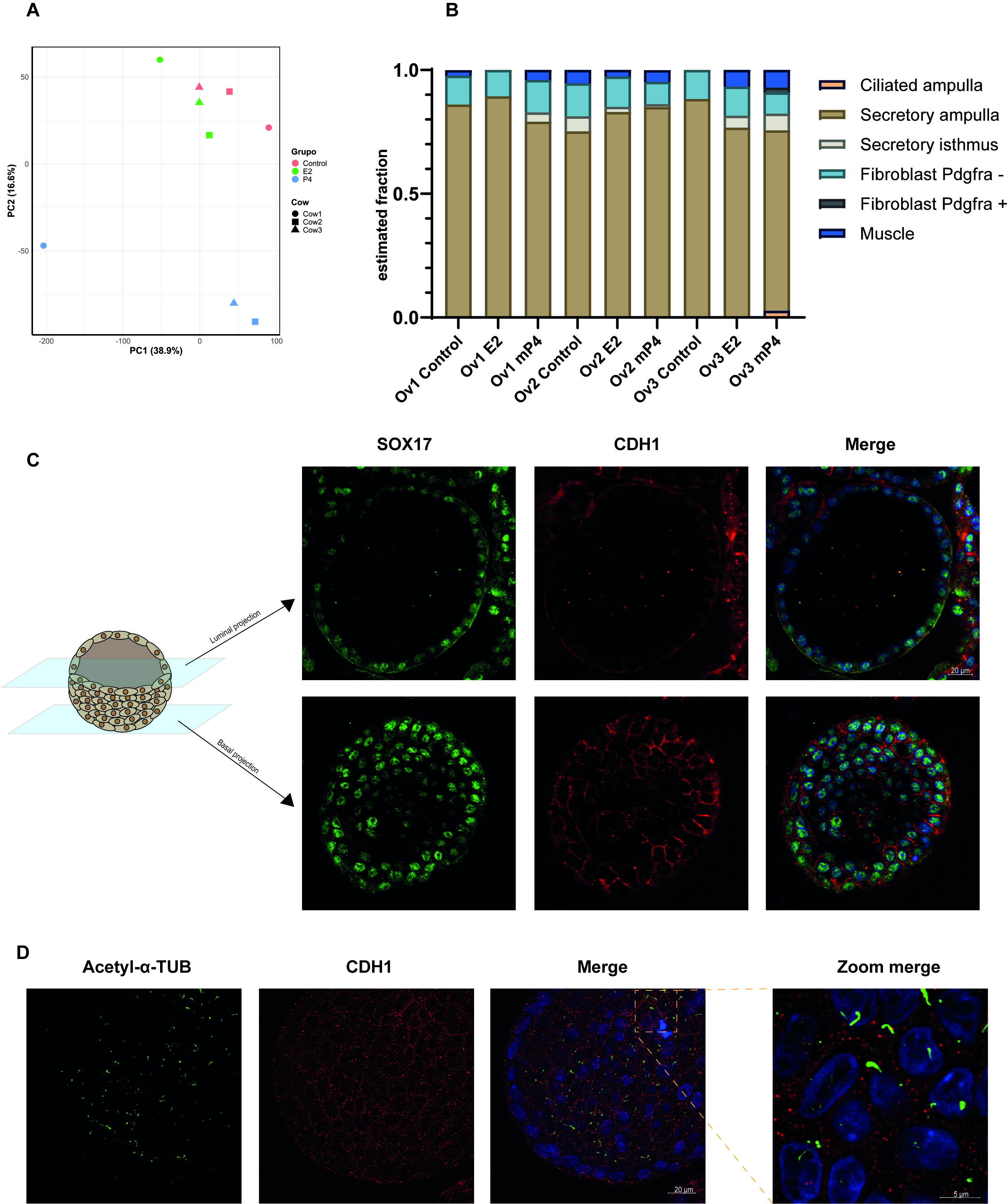

Supplement: Supplementary file 10 — High Resolution Image (TIF 35.0 MB) [file 18_2026_6200_MOESM9_ESM.tif]

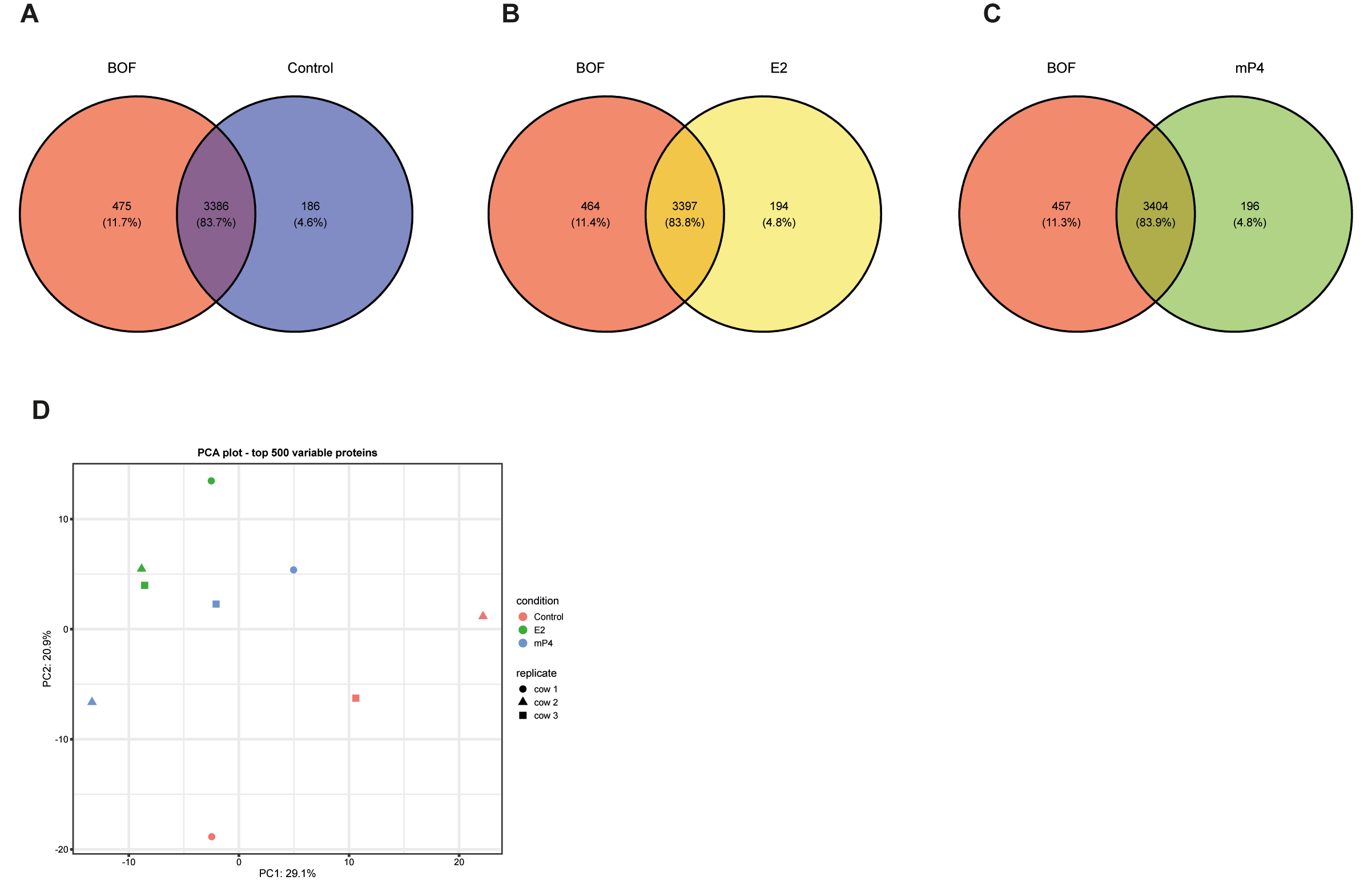

Supplement: Supplementary file 11 — Supplementary Figure 2. (A–C) Venn diagrams showing proteins identified in bovine oviductal fluid from the early luteal phase (BOF) compared with organoid-derived secretions (ODS) from control organoids (A), estradiol-treated organoids (E2, B), and organoids treated with estradiol, medroxyprogesterone, and cAMP (mP4, C). (D) Principal component analysis (PCA) of the proteomic profiles of ODS from the three hormonal treatments [file 18_2026_6200_Fig6_ESM.png]

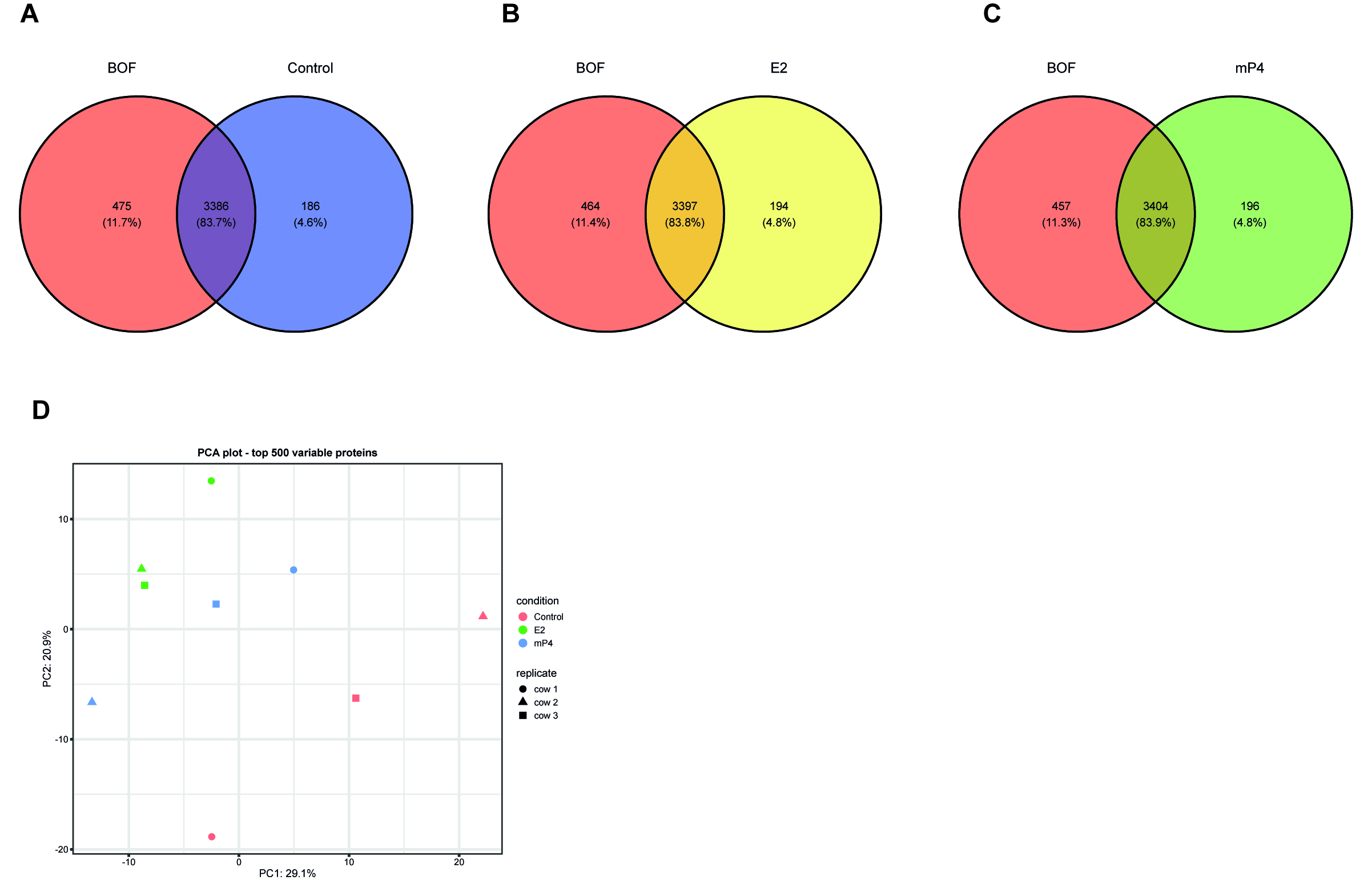

Supplement: Supplementary file 12 — High Resolution Image (TIF 15.5 MB) [file 18_2026_6200_MOESM10_ESM.tif]
